# Airway Management With a GlideScope® Spectrum LoPro Blade in a Child With Giant Facial Rhabdomyosarcoma: A Case Report

**DOI:** 10.1155/cria/3028746

**Published:** 2026-06-03

**Authors:** Morio Kosokabe, Satoshi Sunaga, Ayano Sekigawa, Yukitoshi Niiyama

**Affiliations:** ^1^ Department of Anesthesiology, Akita Kousei Medical Center, Akita, Japan; ^2^ Department of Anesthesiology and Intensive Care Medicine, Akita University Graduate School of Medicine, Akita, Japan, akita-u.ac.jp

## Abstract

Difficult airways can cause severe hypoxia when inducing general anesthesia unless they are well managed, especially for pediatric patients. Successful management of pediatric difficult airways requires meticulous assessments and preparations. The GlideScope® Spectrum LoPro is a thin, hyperangulated blade. Although its utility has been demonstrated in previous studies on Pierre Robin manikins or patients with severely restricted mouth openings, as yet there have been no case reports on its efficacy for children with difficult airways. We encountered a 14‐year‐old female patient with giant facial rhabdomyosarcoma who was scheduled for tumor debulking surgery under general anesthesia. Her mouth opening was limited to 2 child finger‐widths, with Mallampati Classification IV, meaning an anticipated difficult airway. For this anticipated difficult airway, we prepared several airway devices and planned our possible approaches carefully. When industry‐standard options became untenable, we switched to the GlideScope® LoPro blade, completing oral intubation under spontaneous breathing without any complications. This result demonstrated that the GlideScope® Spectrum LoPro blade is useful and effective for at least some forms of difficult airways, with confirmation that it is useful in cases involving giant facial tumors. Its hyperangulated blade offered improved vocal cord visualization. Overall, we have concluded that its use in specialized cases could increase intubation success rates for pediatric patients.

## 1. Introduction

The American Society of Anesthesiologists (ASA) recommends considering an awake or sedated approach for pediatric patients with suspected difficult airways [1]. However, “awake” intubation is frequently impossible in children who cannot understand or cooperate, and the airway must be maintained under sedation or general anesthesia while preserving spontaneous breathing [2].

Rhabdomyosarcoma is the most common soft tissue sarcoma in children. It is highly malignant and may arise in any part of the body [3]. Depending on its size and site, facial rhabdomyosarcoma may increase the difficulty of airway management.

GlideScope® video laryngoscope (Verathon Inc., Bothell, WA, USA) is a type of indirect laryngoscope connected to a monitor that is separated from the blades. The unique shape of the blades contributes to better glottis visualization; however, an altered technique of the blade insertion and the manipulation of oral structures is required [4]. GlideScope® Spectrum LoPro blades are characterized by hyperangulated and thin shapes, and GlideScope Core system offered by Verathon® enables us to see the views from both the GlideScope® LoPro blade and the bronchoscopy on a single monitor. Thus, it is potentially useful for difficult airways in cases where we require both video laryngoscopy and bronchoscopy at the same time. However, no case reports related to pediatric patients have described the use of LoPro blades for difficult airways. We herein present a pediatric case of giant facial rhabdomyosarcoma that was successfully intubated using the LoPro blade after intubation with direct laryngoscopy or McGRATH™ was not considered feasible.

## 2. Case Presentation

The patient was a 14‐year‐old female with a height of 142 cm and weight of 29.5 kg. She was diagnosed with primary rhabdomyosarcoma in the right temporal fossa 10 years ago. Despite undergoing several resections and chemotherapy, the tumor repeatedly relapsed locally on the right side of the face. The tumor kept growing, making it difficult to control tumor bleeding, and she required hospitalization for blood transfusions and pain control.

**FIGURE 1:**
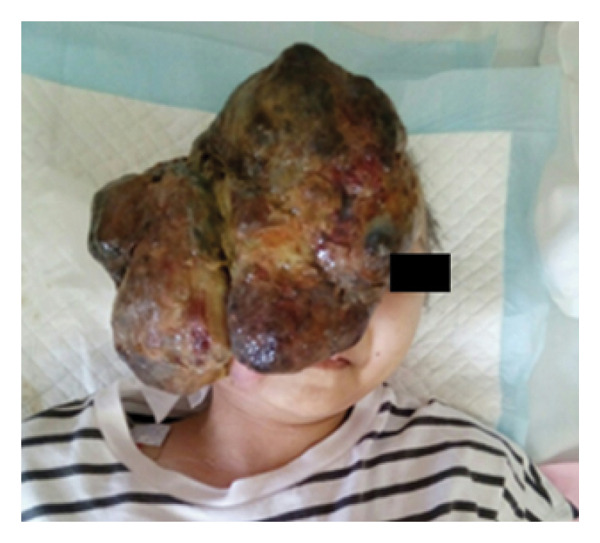
Photograph of the patient prior to the operation.

**FIGURE 2:**
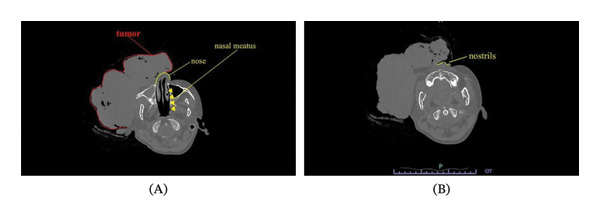
Preoperative CT scans of the patient. (A) The tumor had infiltrated the right orbit and maxillary sinus. The nasal meatus was open, and the upper airway was intact. (B) As the image shows, even though the patient’s nostrils were open, the tumor had grown right in front of the nose, meaning nasal intubation was not feasible.

Tumor debulking surgery was scheduled. The tumor size was 190 × 90 mm and primarily involved the right facial region, covering both nasal cavities and the right corner of the mouth (Figure 1). Her breathing remained stable, and she was able to sleep in a supine position. Figure 2 shows the preoperative CT scan images of the patient. The tumor had infiltrated the right orbit and maxillary sinus, but the upper airway was intact (Figure 2A), and her nostrils were open (Figure 2B). Mouth opening was limited to 2 child finger‐widths, with Mallampati Classification IV, meaning an anticipated difficult airway. We proposed tracheotomy under local anesthesia but did not obtain consent from the patient and her parents. Although her nostrils were open, we determined that nasal intubation was not feasible due to the tumor in front of the nose (Figure 2B). By adjusting the tumor manually from the front to the side of the face, a mask fit well enough to introduce general anesthesia with sevoflurane. Manual ventilation was also feasible in the case of an emergency. We adopted a strategy of airway management under spontaneous breathing to maintain oxygenation during intubation. With the preparation of a GlideScope® Spectrum LoPro single‐use video laryngoscope blade, B Flex™ disposable flexible bronchoscope, GlideScope Core System, and McGRATH™ MAC as well as our Difficult Airway Management cart, we planned to perform intubation with a video laryngoscope as our first approach. Since we had limited experience with fibrotic intubation for pediatric patients, we firstly selected McGRATH^TM^ which we are more familiar with than the GlideScope® LoPro blades. If McGRATH^TM^ did not give us an adequate view, we planned to switch to the GlideScope® LoPro. If we could not detect the glottis with the LoPro blade, we planned to perform bronchoscope‐guided intubation with or without the LoPro blade. If intubation was still not achieved, we planned to cancel or postpone the surgery. If it was not possible to ventilate and intubate the patient, we planned to proceed with emergency tracheotomy by pediatric surgeons.

**FIGURE 3:**
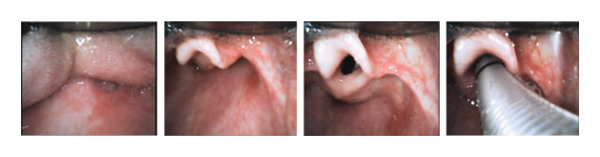
GlideScope® monitor views using the LoPro blade. The left side; the patient’s tongue and root of the uvula. The second left: the patient’s epiglottis. The second right: the patient’s glottis just before intubation. The right side: endotracheal tube insertion.

Anesthesia was induced with 3% sevoflurane following the inhalation of 2 L/min oxygen and 4 L/min nitrous oxide, with the intravenous infusion of 0.4 mcg/kg/h of dexmedetomidine. A 4% lidocaine topical solution was sprayed into the oral cavity with McGRATH™ #2 using Jackson’s spray, a topical anesthesia spray device, and the epiglottis was not visible. We switched to the GlideScope® LoPro S2 blade and applied local anesthesia. We visualized the epiglottis and sprayed local anesthetics into the larynx and trachea with the LoPro blade. The vocal cords were clearly visualized and 6.0 mm of the spiral tube with a stylet was inserted (Figure 3). There were no body movements or harmful reflexes associated with intubation. The time from the initiation of anesthesia to intubation was 20 minutes, and the time from visualizing the glottis with the LoPro blade to intubation was 20 seconds.

**FIGURE 4:**
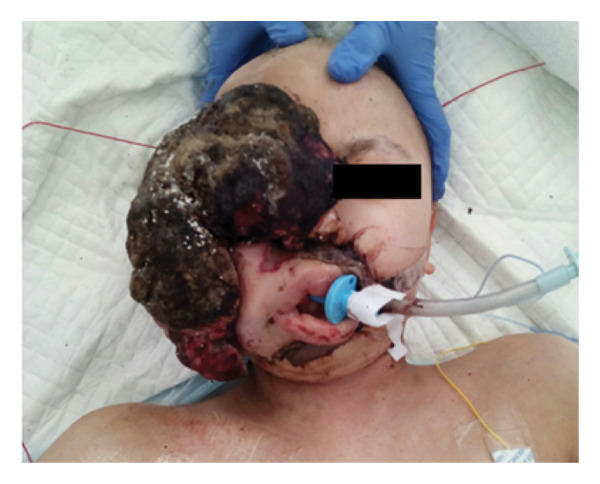
The patient’s picture after surgery. Surgery successfully decreased the tumor size by approximately 50%.

Surgery successfully decreased the tumor size by approximately 50% (Figure 4), and the tumor in front of her mouth was completely eliminated, yet soft tissue edema alongside the upper airway could occur during the surgery. Thus, we considered this case an “at‐risk” extubation under Difficult Airway Society (DAS) extubation guidelines [5]. To be specific, we planned airway exchange catheter‐assisted extubation with the patient fully awake. Since significant bleeding of 1800 mL occurred during the procedure, the patient was transferred to the Intensive Care Unit (ICU) with intubation under sedation for systemic management. The tracheal tube was removed the day after surgery, and the patient was discharged from the ICU on the second postoperative day.

## 3. Discussion

Few studies have investigated the incidence of difficult intubation in pediatric patients [6]. A retrospective study reported pediatric difficult airways in only one out of 542 pediatric patients (0.18%) [7]. We encountered a child with a suspected difficult airway due to a giant facial tumor. Since this was a pediatric case with suspected difficult airways, a meticulous plan to secure the airway and prevent devastating consequences was crucial. We also carefully considered the best airway device for this patient. The GlideScope® LoPro blade seems to be useful and effective for pediatric difficult airways.

Awake intubation is the gold standard technique for anticipated difficult airways in adults, and guidelines have been published [8]. In 2022, ASA renewed practical guidelines for the management of difficult airways [1] and included management for pediatric difficult airways. The use of awake airway techniques for pediatric difficult airways is limited [1, 4]. Since our patient refused to undergo awake intubation or tracheotomy under local anesthesia, the airway strategy to maintain intrinsic airway tone with spontaneous breathing was considered safer than rapid induction, enabling the patient to continuously breathe oxygen during intubation. Slow induction with sevoflurane and the intravenous infusion of dexmedetomidine successfully achieved an appropriately sedated state. Although flexible bronchoscopic intubation has been considered the gold standard for managing anticipated difficult airways, the videolaryngoscope has emerged as a viable alternative [9, 10]. At least one type of video laryngoscope, flexible intubating scope, or rigid or semirigid scope needs to be available for anticipated difficult airway management [11]. Furthermore, the successful management of pediatric difficult airways is facilitated by a preprocedure assessment and preparation [4]. We prepared two types of video laryngoscopes, a flexible bronchoscope, andGlideScope Core System, and planned for the timing of device changes. Specifically, we used McGRATH^TM^ as our first choice, and we could not detect the epiglottis. Then, we switched to the GlideScope® LoPro blade, enabling us to visualize vocal cords. If the LoPro blade did not give us an adequate view, then we would proceed to flexible endoscopic intubation with or without the LoPro blade. This detailed planning contributed to safe and secure intubation.

**FIGURE 5:**
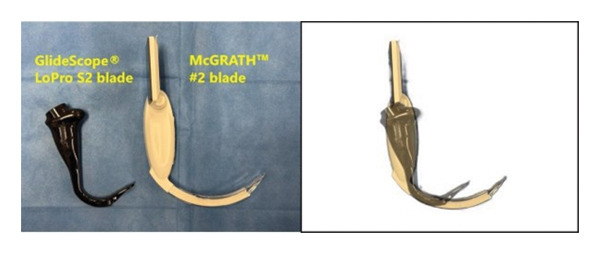
The comparison between GlideScope® LoPro S2 blade and McGRATH™ #2 blade. The blade angle of the LoPro is higher than the McGRATH™, and the blade itself is shorter.

Previous studies showed that GlideScope® is useful in challenging cases. GlideScope® videolaryngoscopy has been associated with improved glottis visualization in patients with difficult airways [12] and fewer intubation attempts than standard Macintosh blades in obese adult patients [13]. GlideScope® also improved glottis visualization and enabled successful intubation in pediatric patients with suspected difficult airways [14]. Moreover, GlideScope® LoPro is a thin and hyperangulated blade (Figure 5). In a manikin study, the LoPro T3 blade showed advantages in dental force, c‐spine motion, and intubation duration over McGRATH^TM^ Mac 4 [15]. Furthermore, LoPro blades provided a good and fast view of the vocal cords in a Pierre Robin manikin [16]. It has also been demonstrated that GlideScope Spectrum^TM^ LoPro blades are useful and safe in patients with significantly restricted mouth opening when performed by an experienced airway provider [17]. A previous study has shown that hyperangulated blades may ensure a good and fast view of the vocal cords although they require specific training to master the technique [16]. In this case, the epiglottis was not visible with McGRATH™ #2, but we detected the vocal cords with the LoPro S2 blade, which was considered to be perfectly fit with this patient. After all, GlideScope® LoPro is an integrated device with a handle and blade, and the video feed from the blade tip can be viewed on an external monitor. The LoPro S2 blade used for this patient has a smaller device body and a slimmer handle (Figure 5), which we hypothesized would be effective to increase the operability of the laryngoscope and detect the vocal cords in patients with difficult airways.

Some kinds of hyperangulated blades are currently commercially available. X blade, a hyperangulated blade for McGRATH™, was only available in size #3 and was not suitable for this case. In the previous study comparing C‐MAC D‐Blade with GlideScope® LoPro which are hyperangulated blades, G. Serocki et al. demonstrated that both D‐Blade and GildeScope® LoPro resulted in an improved view of the glottic opening with successful tracheal intubation in all adult patients with expected difficult airways [18]. On the other hand, according to the study which examined the force exerted on the base of the tongue during intubation in a standard pediatric model, D‐Blade required significantly lower force compared with GlideScope® LoPro [19], meaning C‐MAC D‐Blade might be more tender for pediatric patients especially in case of awake intubation with a videolaryngoscope.

Although videolaryngoscopy with standard blades was recently associated with a significantly higher success rate than videolaryngoscopy with nonstandard blades, including GlideScope LoPro blades, in infants weighing < 5 kg [20], evidence to support the use of hyperangulated blades in cases where standard blades fail, and the airway is difficult and limited; therefore, the next step needs to be an alternative advanced technique including the use of hyperangulated blades with a stylet [11, 16].

Pediatric difficult airways are rare and not encountered by many anesthesiologists. However, healthcare professionals must keep updated with the latest techniques, technologies, and guidelines to ensure the highest standard of care [21]. Moreover, access to hands‐on training with modern airway instruments supports the management of difficult pediatric airways [4].

Further research and case reports, including patients with congenital genetic disorders such as Pierre Robin sequence and Treacher Collins syndrome, are needed to prove whether our approach to this case was appropriate.

## 4. Conclusions

In pediatric patients with difficult airways, a preprocedure assessment and preparation are essential. We demonstrated that the GlideScope® Spectrum LoPro blade was useful for an anticipated difficult airway due to a giant facial tumor.

We presented this case report as a poster discussion at the 72nd annual meeting of the Japanese Society of Anesthesiologists held in Kobe in June 2025. Patient consent was obtained for the presentation at a conference as well as the publication for a case report.

## Funding

No funding was received for this manuscript.

## Conflicts of Interest

The authors declare no conflicts of interest.

## Data Availability

The data that support the findings of this study are available on request from the corresponding author. The data are not publicly available due to privacy or ethical restrictions.
